# Complete remission of thrombotic microangiopathy after treatment with eculizumab in a patient with non-Shiga toxin-associated bacterial enteritis: A case report

**DOI:** 10.1097/MD.0000000000004104

**Published:** 2016-07-08

**Authors:** Taku Omura, Eizo Watanabe, Yasufumi Otsuka, Yoko Yoshida, Hideki Kato, Masaomi Nangaku, Toshiyuki Miyata, Shigeto Oda

**Affiliations:** a Department of Emergency and Critical Care Medicine, Graduate School of Medicine, Chiba University, Chiba; b Department of Pediatrics, Faculty of Medicine, Saga University, Saga; c Division of Nephrology and Endocrinology, Graduate School of Medicine, The University of Tokyo, Tokyo; d Department of Cerebrovascular Medicine, National Cerebral and Cardiovascular Center, Osaka, Japan.

**Keywords:** abdominal infection, acute kidney injury, atypical hemolytic uremic syndrome, membrane cofactor protein, plasma exchange

## Abstract

To describe a case of complete remission of thrombotic microangiopathy after treatment with eculizumab in a patient with non-Shiga toxin-associated bacterial enteritis.

Case report: Medical/surgical intensive care unit (ICU) of a university teaching hospital.

A 62-year-old man presented to a local hospital with mucous and bloody stool persisting for 1 month and worsening abdominal pain for 2 weeks. He had thrombocytopenia and renal dysfunction and was admitted with a diagnosis of sepsis due to intraabdominal infection. However, he did not respond to antimicrobial therapy, and after 7 days he was transferred to the Chiba University Hospital ICU.

Antimicrobial therapy was continued, and continuous hemodiafiltration was initiated on ICU day 3, but the patient's condition deteriorated and he became anuric. Plasma exchange (PE) was initiated on ICU day 11, but anuria and thrombocytopenia persisted. Intravenous eculizumab therapy was initiated on day 26 and resulted in quick recovery of urine output and platelet count and successful discontinuation of renal support.

The diagnosis of thrombotic microangiopathy was established by the presence of schistocytes on the peripheral blood smear on ICU day 9. A plasma sample collected prior to initiation of PE showed a disintegrin-like and metalloproteinase with thrombospondin type 1 motifs member 13 (ADAMTS13) activity level of >10% (25.1%). The absence of both Shiga-toxin producing *E coli* in feces and anti-Shiga-toxin antibody in blood led to suspicion of atypical hemolytic uremic syndrome (aHUS). Genetic test identified a nonsynonymous mutation (p.Ala311Val) in the membrane cofactor protein gene (*MCP*).

Although the pathological significance is currently unknown, this mutation may have been the cause of adult-onset aHUS in our patient. In this case, eculizumab was successfully introduced and discontinued, and the patient remained relapse-free after 1 year of follow-up. The duration of eculizumab therapy for patients with aHUS should be determined on a case-by-case basis and possibly according to the causative genetic mutation, even though discontinuation of eculizumab therapy once initiated is not generally recommended.

## 1 Introduction

Thrombotic microangiopathy (TMA) is a syndrome characterized by systemic microvascular thrombosis and organ failure. Two major variants of TMA are hemolytic uremic syndrome (HUS) caused by Shiga toxin and thrombotic thrombocytopenic purpura (TTP) associated with decreased activity of a disintegrin-like and metalloproteinase with thrombospondin type 1 motifs member 13 (ADAMTS13]). Other etiologies of TMA include infection, drugs, and malignancy. TMA associated with abnormalities in the complement pathway is specifically designated as atypical hemolytic uremic syndrome (aHUS).^[1]^

aHUS has traditionally been treated by plasma exchange and infusion of fresh frozen plasma. However, these supportive therapies provide only transient remission and have been associated with poor long-term prognosis.^[2]^ More recently, the clinical efficacy of eculizumab in the treatment of aHUS has been reported. Eculizumab is a monoclonal antibody that specifically binds the 5th component of the complement (C5) and suppresses excessive complement activation.^[3]^

We herein report complete remission of a refractory case of TMA upon initiation of intravenous infusion of eculizumab in a patient with bacterial enteritis. Findings in the case suggested that the patient had aHUS in association with an abnormality in the complement pathway derived from a recently reported genetic mutation.

## 2 Case report

A 62-year-old man presented to his local hospital after 1 month of mucous and bloody stool and 2 weeks of worsening abdominal pain. He had leukocytosis (1.4 × 10^4^/μL), thrombocytopenia (6.4 × 10^4^/μL), and elevated blood urea nitrogen (BUN; 71 mg/dL) and serum creatinine (2.56 mg/dL) levels. The patient was admitted with a suspicion of sepsis secondary to intraabdominal infection, and broad-spectrum antimicrobial therapy was initiated. However, his condition deteriorated, and after 7 days, he was transferred to the intensive care unit (ICU) at Chiba University Hospital.

On admission to the ICU, the 165.3 cm tall patient weighing 99.3 kg was fully conscious (Glasgow Coma Scale E4V5M6), but he had a tendency to somnolence. His blood pressure was 154/103 mm Hg, heart rate 115/minutes, respiratory rate 30/minutes, body temperature 37.9 °C, and SpO_2_ 96% on room air. The conjunctiva and skin were icteric. The chest was clear to auscultation. There was abdominal distention with mild hypogastric tenderness but no sign of peritoneal irritation. There was pitting edema in the upper and lower extremities. The white blood cell count was 1.2 × 10^4^/μL, C-reactive protein level was 23.9 mg/dL, and procalcitonin level was 8.92 ng/mL, which were consistent with a diagnosis of bacterial infection. The platelet count had dropped to 3.8 × 10^4^/μL, and the prothrombin time was 41% with a fibrin degradation product level of 53.1 μg/mL, indicative of coagulopathy. The lactate dehydrogenase level was 392 IU/L, aspartate transaminase and alanine transaminase levels were 50 and 17 IU/L, respectively, and also total and direct bilirubin levels were 6.2 and 4.6 mg/dL, respectively; however, other indicators of hemolysis, including anemia and schistocytosis, were absent. The BUN had increased to 92 mg/dL and the creatinine level was stable at 2.09 mg/dL. The CH50 level was 40.6 U/mL (normal range: 30–50 U/mL), the C3 level was 85 mg/dL (normal range: 65–135 mg/dL), and the C4 level was 23 mg/dL (normal range: 13–35 mg/dL).

**Figure 1:**
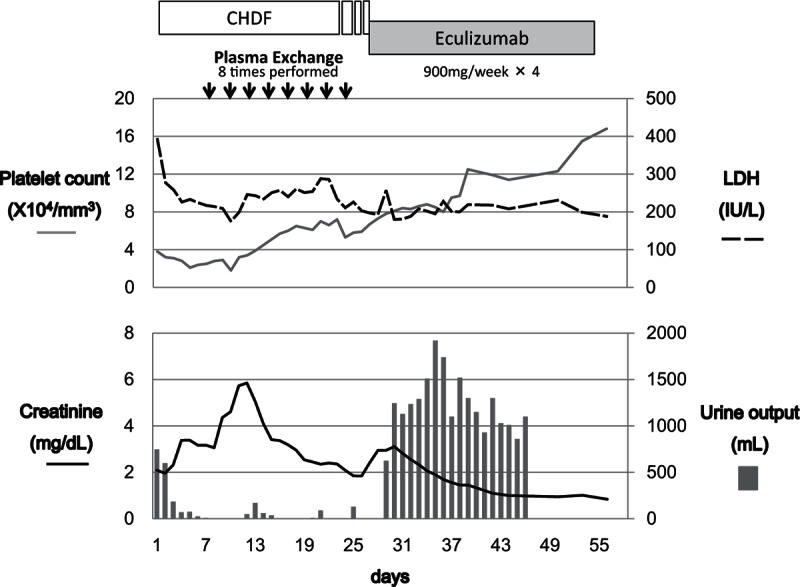
CHDF was introduced on ICU day 3. The patient remained oliguric after 8 sessions of PE therapy. Eculizumab was introduced to address a decrease in urine output on ICU day 26 in suspicion of aHUS, and a dramatic increase in urine output was observed on the next day. Renal support was successfully discontinued on the same day. aHUS = atypical hemolytic uremic syndrome, CHDF = continuous hemodiafiltration, ICU = intensive care unit, PE = plasma exchange.

The patient's clinical course is summarized in the Fig. 1. Since a stool culture test on ICU admission identified *Enterococcus* species and *Corynebacterium striatum*, a diagnosis of severe sepsis derived from bacterial enteritis was established and wide-spectrum antimicrobial therapy was initiated. Continuous hemodiafiltration was initiated on ICU day 3 but oliguria progressed to anuria, thrombocytopenia persisted, and laboratory values did not improve. Blood, urine, and spinal fluid cultures taken on admission to the ICU were all negative, which eliminated the possibility of additional foci of infection. Two polyps (one each in the sigmoid colon and the rectum) were discovered on lower gastrointestinal endoscopy, which were diagnosed at histopathology as early-stage nonhemorrhagic adenocarcinoma. The polyps did not appear to be the cause of the bloody stool. Additional laboratory evaluations were negative for other hematological disorders or connective tissue diseases. On ICU day 9, schistocytosis (0.5%) was noted on the peripheral blood smear, and a diagnosis of TMA was established. The Hct level also had dropped to 23.5% on the same day and the declining trend continued, therefore red blood cell transfusion was performed days later when he complained dyspnea. Plasma exchange (PE) therapy was initiated on ICU day 11. The patient had only a slight remission of thrombocytopenia after a total of 8 PE sessions (2.1 × 10^4^/μL before the 1st PE and 5.9 × 10^4^/μL after the 8th PE), but there was no notable clinical improvement and the anuria persisted (Fig. 1). The level of ADAMTS13 activity in a plasma sample collected from the patient prior to initiation of PE was 25.1%, and the sample was negative for ADAMTS13 inhibitor. These findings eliminated the possibility of TTP. Furthermore, the stool was negative for Shiga toxin-producing-strains of *Escherichia coli*, and the serum titer for anti-Shiga-toxin antibody was also negative, eliminating the possibility of HUS. Although we considered TMA secondary to colorectal cancer in the differential diagnosis, the endoscopic findings of intramucosal carcinoma and negativity for tumor markers eliminated this possibility. Careful consideration of other possible causes, previous history, and drug history failed to identify an extrinsic etiology of TMA in this patient, and we ultimately suspected aHUS as the cause. Accordingly, intravenous eculizumab treatment (900 mg, once a week) was initiated on ICU day 26. An increase in urine output was observed on the next day, and renal support was successfully discontinued on the same day. And also marked recovery of the platelet count was obtained in combination with the effectiveness of supportive therapy including PE. Biweekly eculizumab infusion was repeated 4 times and then discontinued.

After approval by the institutional review boards of Chiba University, the University of Tokyo, and National Cerebral and Cardiovascular Center, an anticomplement factor H (CFH) antibody assay and an analysis of complement factor H-related (CFHR) genes were performed using a blood sample collected from the patient prior to initiation of PE. Also written informed consent was obtained from the patient before the assays. The anti-CFH antibody level was 6.16 AU/mL, which indicated negativity for anti-CFH antibody. We performed the multiplex ligation-dependent probe amplification (MLPA) analysis to screen the copy numbers of *CFHR1/3* on chromosome 1q32 using a commercially available kit (MLPA kit P236-A2; MRC-Holland, The Netherlands),^[4]^ and found that *CFHR1/3* did not show gene deletions. However, moderate hemolysis was induced in concomitant hemolytic assays using sheep red blood cells (RBCs), and analysis for mutations potentially associated with aHUS identified a nonsynonymous mutation (p.Ala311Val) in the gene encoding membrane cofactor protein (*MCP*). All screened genes are presented in the Table 1.

**Table 1:**
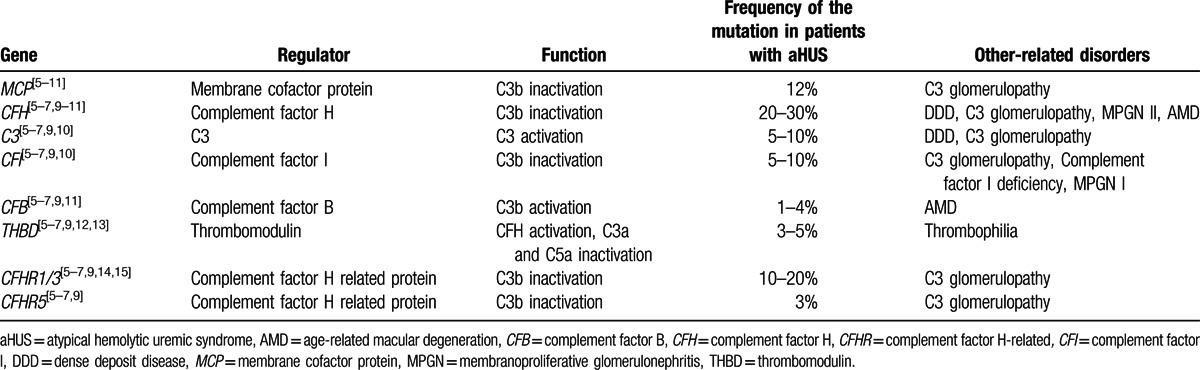
Screened genes and clinical phenotypes.

The patient was discharged from the ICU on day 37 and was discharged to home on day 58. He was followed up regularly as an outpatient and was receiving 40 mg/day of prednisolone. At approximately 1 year after discharge, the patient remained in stable condition without relapse or resumption of eculizumab therapy.

## 3 Discussion

aHUS is a peculiar type of TMA that develops in association with endothelial dysfunction and platelet surface activation induced by dysregulation and chronic activation of the alternative complement pathway. Familial HUS caused by abnormalities in the gene encoding *CFH* was first reported in 1998^[16]^ and since then additional genes that may be associated with aHUS have been identified. In our case, a patient with infectious enteritis developed TMA. We suspected aHUS after excluding HUS, TTP, and secondary TMA. As PCR studies were not performed for this case thorough his disease course and antibody screening for *Shiga* toxin-producing *E coli* was only performed at admission, it is undeniable that our examination was not enough to eliminate the possibility of *Shiga* toxin-producing *E coli* infection for this case. However, the responsiveness of PE and also the effectiveness of eculizumab administration were obvious enough to diagnose this case as complement mediated aHUS.

Plasma C3, C4, complement factor B (CFB) levels, anti-CFH antibody titers, and measurement of MCP expression by leukocytes are recommended for the diagnosis of aHUS.^[1]^ In the present case, the patient was negative for anti-CFH antibody and had no gene deletions in *CFHR1* and *CFHR3*. However, moderate hemolysis in a hemolytic assay using sheep RBCs as an auxiliary diagnostic method suggested an abnormality in the alternative complement pathway,^[17,18]^ and a nonsynonymous *MCP* mutation, Ala311Val,^[18]^ was detected. Mutations in *MCP* have previously been associated with aHUS. This patient has no family history of aHUS, suggesting that the mutation may not have been inherited. The minor allele frequency for this novel mutation has not been extensively compared with that in nonaffected individuals. Therefore, it is categorized as a “variant of uncertain significance.”^[19]^

MCP is a membrane-bound protein that serves as a cofactor of complement factor I (CFI) and facilitates CFI-mediated cleavage of C3b expressed on the same cell membrane.^[5]^ Since C3b is capable of binding to and injuring pathogens as well as autologous cells, rapid cleavage of C3b protects autologous cells from injury by the complement system.^[5]^ Development of aHUS secondary to *MCP* mutation involves a loss of function of the MCP that leads to failed C3b inactivation and the resulting chronic activation of complement.

Abnormalities in *MCP* have been reported in approximately 10% to 20% of patients with aHUS.^[2]^ The *Ala311Val* point mutation is found within the serine–threonine–proline-enriched domain of *MCP*. Up to 90% of patients with aHUS who have abnormalities in regions of *MCP* encoding parts of the transmembrane domain or any of the 4 complement control protein (CCP) domains recover after PE or supportive care treatment alone, and renal support is successfully terminated in most of these cases.^[6,20]^ Although an amino acid substitution in MCP suggestive of an *MCP* mutation causing aHUS was discovered in our patient, he did not respond well to plasma exchange.

Eculizumab has been effective in patients with aHUS irrespective of their response to PE therapy,^[21]^ and in the present case, the initiation of eculizumab induced a rapid increase in urine output, allowing successful withdrawal of renal support in combination with plasma therapy, etc. Criticism that the initiation of eculizumab just coincide with resolution of the disease may arise because the platelet count was gradually rising prior to start of diuretic phase. However, the eculizumab infusion must have at least triggered the occurrence because the period of anuria was fairly long even though we continued PE regularly and the diuretic phase came suddenly following administration of eculizumab. Progression to end-stage renal failure (ESRF) is a key determinant of survival in patients with aHUS, and systemic complications of aHUS are serious.^[2]^ In 2 small open-label prospective trials, up to 80% of patients with aHUS were able to discontinue renal support after initiation of eculizumab therapy. As approximately 30% (5 in 18) of the patients who missed eculizumab doses experienced severe subsequent complications of TMA, discontinuation of eculizumab was not favored.^[3]^ However, cases of aHUS associated with *MCP* abnormalities seem to have a more favorable prognosis. Accordingly, in the present case, we decided to discontinue the eculizumab treatment after 4 weekly doses, and there was no deterioration in renal function during 1 year of follow-up. This suggests a possibility that safe and successful discontinuation of eculizumab is expected in mild aHUS caused by *MCP* mutations. We understand the exact association of the *Ala311Val* mutation and pathogenesis of aHUS is still opaque. Actually, this case is adult-onset aHUS, therefore carriers of the mutation are suggested to pose potential risk to cause the disease by severe infection, that is, septic insult as this case had. As aHUS is an extremely rare disease, with an annual incidence as low as 2/1,000,000 in adults and 7/100,000 in children, continued accumulation of knowledge regarding patterns of disease onset and response to treatments under different genetic backgrounds will be essential for the development of future treatment strategies.^[22,23]^

## 4 Conclusions

We encountered a case of TMA in a patient with bacterial enteritis. The patient had an amino acid substitution in MCP, suggesting that the TMA was due to aHUS associated with a mutation in *MCP*. However, this genetic variant has not yet been proven to be disease causing and its frequency in the general population has not yet been fully studied. Therefore, further genetic testing using a larger patient cohort is needed. The patient had a complete remission of TMA after initiation of intravenous eculizumab therapy, which was sustained as of 1 year after discontinuation of treatment. The present case demonstrates that the duration of eculizumab therapy for patients with aHUS should be determined on a case-by-case basis and possibly according to the causative genetic mutation.
